# Special Issue: Viral Infections in Companion Animals

**DOI:** 10.3390/v14020320

**Published:** 2022-02-04

**Authors:** Margaret J. Hosie, Regina Hofmann-Lehmann

**Affiliations:** 1 Centre for Virus Research, Medical Research Council-University of Glasgow, Glasgow G61 1QH, UK; 2 Clinical Laboratory, Department of Clinical Diagnostics and Services, and Center for Clinical Studies, Vetsuisse Faculty, University of Zurich, CH-8057 Zurich, Switzerland

Companion animals, such as cats, dogs, horses and exotic species, play an important role in society; more than 600 million cats and 900 million dogs live closely with humans worldwide. A ‘One Health’ approach recognises the intimate connections that exist between the health of humans, their companion animals and our shared environment. The emergence of infectious diseases such as COVID-19 reminds us of the importance of identifying and understanding these connections to control and prevent disease. The bond between humans and their companion animals can be strong and lifelong, and the benefits of the human–companion animal bond to human mental and physical health are well recognised. Hence, we see an increasing research focus on viruses that cause disease in companion animals and consequently affect the health and/or well-being of their human guardians. Indeed, research into viral infections of companion animals has led to significant improvements in animal health and the development of effective viral vaccines against important veterinary pathogens. Such developments protect valued companion animals from the consequences of infectious diseases and can contribute to the development of novel and improved methods for the development of diagnostics and vaccines for diseases in humans caused by related viruses. 

For example, in the field of retrovirology, no human retrovirus has been tackled as effectively as feline leukemia virus (FeLV), for which effective vaccines have markedly reduced the prevalence of infection. Studies on cats naturally infected with FeLV have significant comparative value, improving our understanding of disease outcomes following retroviral infection [1,2,3]. A striking feature of the active suppression of FeLV in infected animals is the clearance of latent infection. A major scientific challenge is the elimination of viral reservoirs in HIV-infected individuals, where the report of cats clearing latent FeLV infections is highly significant [4]. 

Veterinary vaccines for coronaviruses that cause important diseases in pigs, chickens, dogs and cats informed the development of human COVID-19 vaccines. Clearly, an improved understanding of the coronaviruses of companion animals [5] and their capacity for cross-species transmission will improve prevention and control strategies for future coronaviruses emerging in animals and humans. Some veterinary coronavirus vaccines have only short-term efficacy following the emergence of new variants, and, similarly, novel SARS-CoV-2 variants require us to monitor vaccine efficacy and potentially update vaccines, based on either emerging variants or variants that induce broad protection. Similar issues have been investigated for feline calicivirus, another RNA virus that mutates rapidly. It is promising that, although a modified-live FCV vaccine based on a variant that was isolated several decades ago did not induce humoral immunity against a recently circulating variant, cellular immunity was induced [6].

The significance of new feline viruses that were recognised recently remains unclear, but a potential link between renal and lower urinary tract disease and feline morbillivirus infections has been highlighted [7], and the identification of two novel feline astroviruses [8] underlines the importance of further studies to better understand companion animal viral infections. It is evident that, with the recognised zoonotic potential of some animal viruses, companion animal virology has great One Health significance. 

Research in the authors’ laboratories is supported by UKRI-BBSRC (MJH) and the Federal Food Safety and Veterinary Office and Federal Office of Public Health of the Swiss Confederation (RHL).
